# Feasibility and Tumor Dynamics of Daily MRI‐Guided Online Adaptive Radiotherapy for Brain Glioma

**DOI:** 10.1002/cns.70905

**Published:** 2026-04-27

**Authors:** Shouliang Ding, Xiumao Yin, Mengqi Sun, Hongdong Liu, Ying Wang, Biaoshui Liu, Mengke Qi, Yuchuan Zhou, Xiaojing Du, Meiling Deng, Wanming Hu, Xiaoyan Huang, Zihuang Li, Yonggao Mou, Yuanyuan Chen

**Affiliations:** ^1^ Department of Radiation Oncology, State Key Laboratory of Oncology in South China Guangdong Provincial Clinical Research Center for Cancer, Sun Yat‐Sen University Cancer Center Guangzhou China; ^2^ United Laboratory of Frontier Radiotherapy Technology of Sun Yat‐Sen University & Chinese Academy of Sciences Ion Medical Technology co., Ltd Guangzhou China; ^3^ Cancer Center The Tenth Affiliated Hospital, Southern Medical University (Dongguan People's Hospital) Dongguan China; ^4^ Department of Radiation Oncology Shenzhen People's Hospital Shenzhen China; ^5^ Department of Pathology, State Key Laboratory of Oncology in South China, Guangdong Provincial Clinical Research Center for Cancer Sun Yat‐Sen University Cancer Center Guangzhou China; ^6^ Department of Neurosurgery, State Key Laboratory of Oncology in South China, Guangdong Provincial Clinical Research Center for Cancer Sun Yat‐Sen University Cancer Center Guangzhou China

**Keywords:** adaptive radiotherapy, brain glioma, feasibility, MR‐Linac, tumor dynamics

## Abstract

**Background:**

Magnetic resonance image (MRI)‐guided radiotherapy can optimize the therapeutic outcomes of brain glioma patients, as it adjusts to tumor changes in the course of radiation treatment. This study evaluates the dynamic changes of tumors and the feasibility of implementing MRI‐guided online adaptive radiotherapy (MRIgOART) for the treatment of brain glioma.

**Patients & Methods:**

This observational prospective cohort study involved patients with brain glioma treated using 1.5 T MR‐Linac from 2021 to 2023. MRIgOART can correct treatment errors and evaluate treatment response through adapt‐to‐position (ATP) and adapt‐to‐shape (ATS) strategies. Dice similarity coefficient (DSC), absolute/relative volume (Vrel), and Hausdorff distance (HD) metrics were used to quantify tumor changes. The covariables subjected to evaluation included: surgical resection extent, 1p/19q status, telomerase reverse transcriptase (TERT) mutation status, O6‐methylguanine‐DNA‐methyltransferase (MGMT) methylation status, and isocitrate dehydrogenase (IDH) mutation status. ART and non‐ART treatment plans were comparatively analyzed based on target coverage and dose constraints for normal brain tissue. The pattern of failure, as the primary endpoint, was evaluated in this study. Secondary endpoints of the study consisted of overall survival (OS) and progression‐free survival (PFS), assessed according to treatment schedules.

**Results:**

The cohort comprised 57 patients. The patients with an interval longer than 10 days from simulation to the Fx1 exhibited more significant tumor changes (*p* < 0.001). The tumor volume showed a gradual reduction during the treatment, whereas the alterations in its location and shape became increasingly evident over time. Multivariate analyses identified associations between prognosis and HD, in addition to a relationship between the extent of surgical resection and DSC. ATS was utilized in 52.6% of patients at least once during treatment, with a higher frequency in TERT wild‐type patients (*p* = 0.013). MRIgOART treatment plans achieved superior target conformality, adequate coverage, and effective sparing of OARs. High‐grade glioma (HGG) patients exhibited median PFS of 13 months (95% CI, 10.2–15.8 months) and OS of 28 months (95% CI, 23.3–32.7 months). Failure analysis revealed 58.9% in‐field, 17.6% marginal, and 23.5% distant recurrences, with IDH mutation status associated with failure patterns.

**Conclusion:**

Preliminary findings in patients with HGG suggest a lower incidence of recurrences within the radiation field and indicate promising outcomes associated with MRIgOART. However, these observations require further validation through comparative studies.

## Graphical abstract

The apparent tumor dynamics were observed during chemoRT in patients with brain glioma. MRIgOART not only corrected daily anatomical variations but also enabled real‐time assessment of treatment response, thereby facilitating dynamic optimization of concomitant therapy protocols. Preliminary findings in patients with HGG suggest a lower incidence of recurrences within the radiation field and indicate promising outcomes associated with MRIgOART.

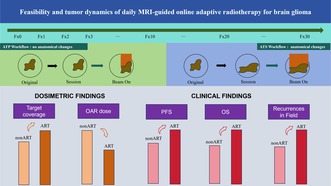

## 1 Introduction

Treatment for brain glioblastoma remains a significant challenge, with the overall survival (OS) rate at 5 years lingering around 5% despite intensive therapeutic approaches [1]. The current standard initial treatment involves fractionated radiotherapy (RT) combined with temozolomide (TMZ) following surgical resection [1, 2, 3]. Magnetic resonance imaging (MRI) serves a critical function in delineating the tumor region and detecting organs at risk (OARs) [4, 5]. To evaluate the response of treatment, clinical practice typically includes an MRI before and after surgery and/or before RT, with a subsequent MRI approximately one month after completing chemoradiotherapy (chemoRT), totaling an interval of roughly 3 months [6]. Nonetheless, the regular monitoring of tumor status via MRI throughout the chemoRT course is not standard practice. While acquiring standalone MRIs during RT is possible, high‐frequency imaging is limited by its feasibility. A limited number of studies have concentrated on MRI throughout the radiotherapy course, and these investigations have generally been restricted to one individual imaging time point within the six‐week RT period [7, 8, 9].

In conventional radiotherapy, daily cone beam computed tomography (CBCT)‐guided RT utilizes bony registration to ensure precise treatment delivery, yet it fails to provide visualization of soft‐tissue tumors [6]. Without an MR image, imaging during chemoRT is limited by the low soft tissue contrast inherent in CBCT scans. Recently developed MR‐Linac systems enable daily soft tissue imaging concurrent with RT, facilitating the monitoring of early brain and tumor changes during chemoRT. Moreover, MRI‐guided online adaptive radiotherapy (MRIgOART), integrated with MR‐Linac systems, enables real‐time adjustment for anatomical variations. This approach potentially allows for reduced treatment margins, which decreases exposure to normal brain tissue while increasing the dose delivered to the target area. Consequently, MRIgOART holds significant potential to advance radiotherapy by improving healthy tissue protection and optimizing disease management, though further robust clinical evidence is essential for definitive validation [10]. Our institution has dedicated efforts to developing a treatment strategy for brain glioma utilizing a 1.5‐T MR‐Linac system (Elekta AB, Stockholm, Sweden). Our study evaluates the continuous dynamic changes of tumors during the six‐week fractionated radiotherapy through online MRI performed at each treatment session. Comprehensive analyses of tumor dynamic factors and their prognostic implications are also provided. Finally, we assess the oncological outcomes and feasibility of MRIgOART in patients diagnosed with high‐grade glioma (HGG).

## 2 Methods and Materials

### 2.1 Study Cohort

This study involved brain glioma patients who underwent treatment with a 1.5‐T MR‐Linac during the period from 2021 to 2023. Ethical approval was obtained from the Research Ethics Committee before study initiation, with all participants providing written informed consent. All patients received daily MRIgOART, with a total dose of 54–60 Gy in 30 fractions. Patients were evaluated through routine clinical examinations and serial MRI scans performed at least every 3 months.

### 2.2 Patient Treatment Workflow

**FIGURE 1:**
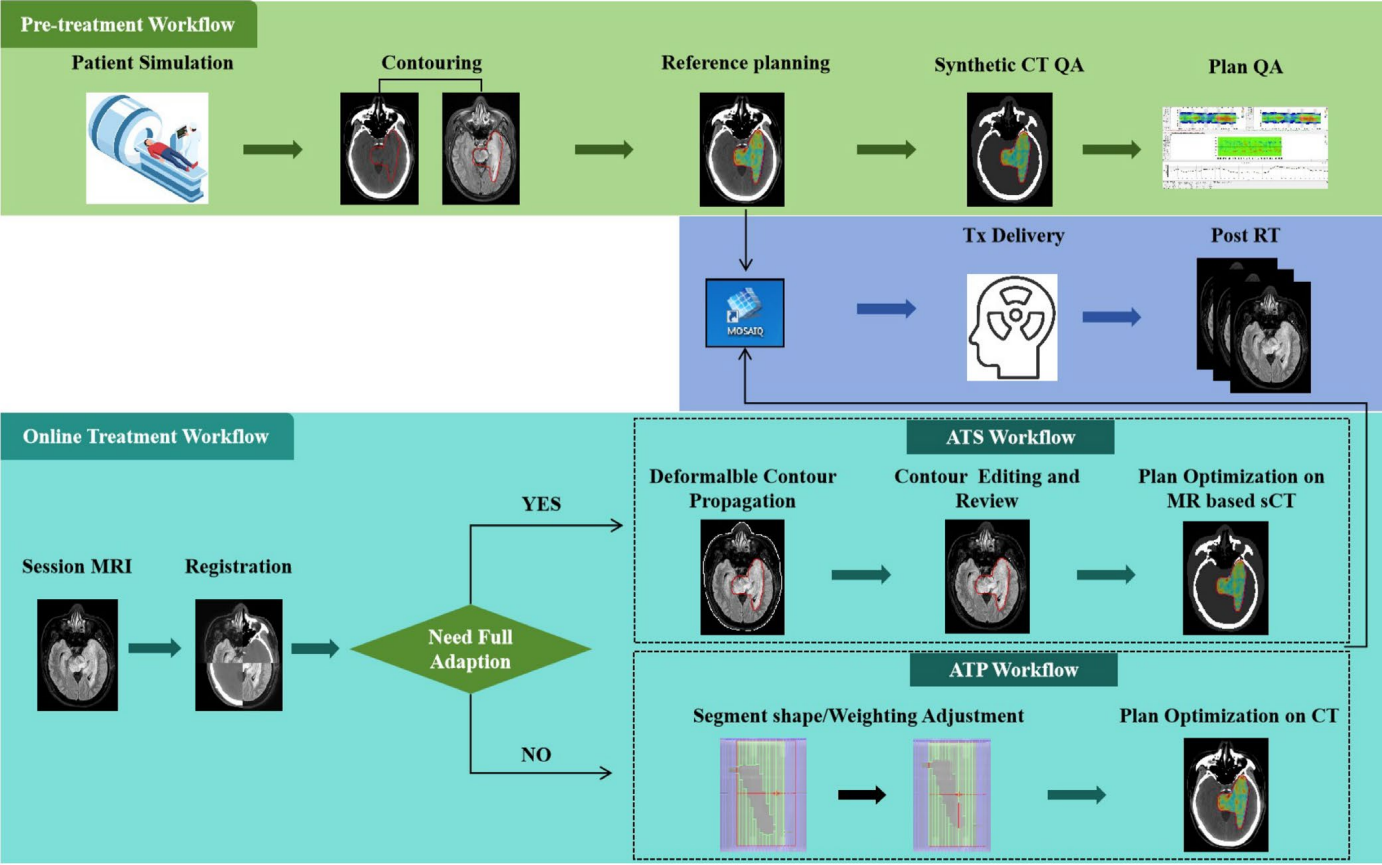
The flowchart for pre‐treatment and online treatment workflow of MR‐Linac.

The patient treatment workflow was summarized in Figure 1. For pre‐treatment reference RT planning, the target volume was delineated on T2‐weighted fluid attenuation inversion recovery (FLAIR) and gadolinium‐enhanced T1‐weighted (T1c) MRI scans obtained through MR simulation (Ingenia MR‐RT, Philips, Netherlands). These images were fused with planning CT scans in accordance with established guidelines. The gross tumor volume (GTV) was identified as the surgical cavity along with any residual tumor that showed enhancement on T1‐weighted imaging [11]. To delineate clinical target volume (CTV), a consistent 1.5 cm isotropic expansion was implemented around the GTV, with due consideration for anatomical boundaries. Subsequently, planning target volume (PTV) may be generated by implementing a 3‐mm isotropic outward expansion for both the GTV and CTV individually. The margin is based on ESTRO‐EANO guideline on target delineation and radiotherapy details for glioblastoma [12].

Initial reference treatment plans were created using the intensity‐modulated radiotherapy (IMRT) approach. The prescribed total dose was either 54 to 60 Gy delivered in 30 fractions to the PGTV. The RT plans were designed to ensure that at least 95% of the PTV volume received the prescribed dose, with 99% of the GTV and CTV also covered, and the maximum dose was restricted to no more than 110% of the prescription. Dose restrictions for OARs were based on the guidelines from ICRU Report 83 [13] as well as institutional standards regarding dose constraints in glioma radiotherapy planning.

For each treatment fraction, the volumetric FLAIR MRI scan was acquired using 3D fast spin echo acquisition in steady state (TSE), a refocus angle of 40°, echo time (TE) of 390 ms, repetition time (TR) of 4800 ms, inversion time (TI) of 1650 ms. The selection between the ATP and ATS techniques was made by oncologists based on the MRI findings acquired on the day of the treatment fraction. In the absence of morphological changes in the tumor or OARs, the ATP technique was used to rectify setup errors through adjustments to the segment shape or weight within the reference treatment plan. In the event of morphological alterations in the tumor or OARs, the ATS technique was implemented to generate an adaptive treatment plan based on the current MRI data, aimed at rectifying deformation‐related discrepancies and safeguarding the precision of the delivered dose. ATS is generally used when significant tumor responses or anatomical changes are observed during treatment.

### 2.3 Tumor Dynamic

The tumor dynamics were evaluated relative to baseline value at Fx0 and consisted of the following parameters.

1. The relative volume (V_rel_) of the target was calculated by dividing the absolute volume of the target at each treatment fraction (Fx1, Fx2, …, Fx30) by the initial target volume obtained at Fx0. A V_rel_ value less than 1 indicates a reduction in tumor size, whereas a value greater than 1 suggests growth.
2. The Dice similarity coefficient (DSC) was used to quantify the degree of volumetric overlap between the target at a given treatment fraction (FxN) and the baseline (Fx0). The DSC is calculated as DSC = 2(V_A_ ∩ V_B_) / (V_A_ + V_B_). The range of the DSC is 0 to 1, where higher values denote a greater degree of overlap between the target volume and the measured volume.
3. Hausdorff distance (HD) provided the maximum extent of discrepancy between the reference and the compared contours. HD is defined as HD (A,B) = max [h(A,B), h(B,A)],$h\left(A,B\right)=\max_{a\in A}\min_{b\in B}\left\|a-b\right\|$.

### 2.4 Covariables

The covariates were systematically evaluated for their association with tumor dynamics during radiotherapy and encompassed the following parameters: extent of surgical resection (Subtotal resection [STR] was defined as the presence of any detectable contrast‐enhancing residual tumor lesions on early postoperative MRI scans, whereas gross total resection [GTR] was defined as the complete absence of identifiable contrast‐enhancing residual tumor tissue on early postoperative MRI examinations), time from surgery to Fx0, time from simulation to Fx1, and molecular characteristics encompassing 1p/19q status (noncodeletion [noncodel] or codeletion [codel]), O^6^‐methylguanine‐DNA‐methyltransferase (MGMT) promoter methylation status (methylated [methy] or unmethylated [unmethy]), isocitrate dehydrogenase (IDH) mutation status (mutated [mut] or wild‐type [WT]), and telomerase reverse transcriptase (TERT) mutation status (mutated [mut] or wild‐type [WT]). The classification of surgical extent was determined through the combination of operative reports with postoperative MRI findings. Additional variables included in the survival analyses were age, sex, and the presence of pseudo‐progression observed during follow‐up, as assessed through MRI and clinical evaluation.

### 2.5 Dosimetric Assessment of ART Treatment Plan

The online adaptive radiotherapy (ART) plans were generated based on the MRI acquired at the time of the current treatment fraction. The contours and electron density data derived from the original reference treatment plan were transferred to the contemporaneous MR image, which was subjected to contour modification and confirmation by attending physicians, followed by re‐verification of the contouring results and electron density map by medical physicists to ensure accuracy. The ART plans were systematically revised and re‐optimized on the basis of the established reference treatment plans, with the aim of aligning with the dynamic anatomical status of the patient during each individual radiotherapy session. In contrast, non‐ART plans were generated by recalculating dose distributions based on the original reference plans without adjusting for daily anatomical changes. For each adaptive ART treatment fraction, a comprehensive comparison was conducted between the ART and non‐ART plans, focusing on target coverage and the dose delivered to OARs.

### 2.6 Oncological Outcome

The oncological outcomes of interest were overall survival (OS), progression‐free survival (PFS), and patterns of failure. Tumor recurrence was defined in accordance with the Response Assessment in Neuro‐Oncology (RANO) criteria for tumor progression [14]. Tumor recurrence patterns were defined based on the relative position between the recurrent tumor area on post‐treatment MRI and the 95% isodose line area in the radiotherapy plan. They were classified into three types: “in‐field” was defined as more than 80% of the tumor area on T1‐weighted contrast‐enhanced images being covered by the 95% isodose line; “marginal” was defined as 20% to 80% of the tumor area on T1‐weighted contrast‐enhanced images being covered by the 95% isodose line; and “distant” was defined as less than 20% of the tumor area on T1‐weighted contrast‐enhanced images being covered by the 95% isodose line.

### 2.7 Statistical Analyses

Categorical variables were presented as frequencies and percentages, whereas continuous variables were reported as medians accompanied by ranges or interquartile ranges (IQR). The normality of the data was evaluated using both graphical methods (histograms and Q‐Q plots) and a formal statistical test. Specifically, the Shapiro–Wilk test was used for all continuous variables. Student's *t*‐test was used to analyze tumor dynamics, target covariates, and dosimetric analysis, with the Mann–Whitney U test utilized as appropriate. For categorical variables, comparisons among different groups were made using Fisher's exact test or X^2^ test as suitable. Pearson's correlation analysis was systematically applied to assess the relationship between the covariates and tumor dynamics. PFS and OS were analyzed using the Kaplan–Meier method, where the time‐to‐event was defined as the interval from the date of surgery to the occurrence of the predefined endpoint or the last follow‐up evaluation. All statistical analyses were two‐tailed, with *p* < 0.05 deemed indicative of statistical significance. Statistical analyses were performed on the SPSS system (Version 31.0, IBM Corp., Armonk, NY, USA) and open‐source statistical software R version 4.0.3 (R Core Team 2020, R Foundation for Statistical Computing).

## 3 Results

This study enrolled a total of 57 patients. The baseline characteristics of the study population are summarized in Table 1. There were 51 (89.5%) HGG patients and 6 (10.5%) low‐grade glioma (LGG) patients. A total of 30 patients (52.6%) underwent ATS adaptive radiotherapy.

**TABLE 1:** Baseline characteristics of the study cohort (*n* = 57).

Age (years), median (range)	44 (21–68)
Sex *n* (%)	
Male	34 (59.6%)
Female	23 (40.4%)
Resection status, *n* (%)	
STR	26 (45.6%)
GTR	31 (54.4%)
Type of tumor, *n* (%)	
HGG	51 (89.5%)
LGG	6 (10.5%)
Bevacizumab, *n* (%)	1 (1.8%)
Time from surgery to planning MRI (days), median (range)	32 (17–107)
Time from simulation to Fx1 (days), median (range)	11 (6–25)
MGMT methylation, *n* (%)	
Methylated	28 (49.1%)
Unmethylated	24 (42.1%)
Unknown	5 (8.8%)
IDH status, *n* (%)	
Mutated	23 (40.4%)
Wild‐type	33 (57.9%)
Unknown	1 (1.7%)
1p/19q status, *n* (%)	
Codeletion	12 (21.1%)
Noncodeletion	45 (78.9%)
TERT status, *n* (%)	
Mutated	31 (54.4%)
Wild‐type	20 (35.1%)
Unknown	6 (10.5%)
ART workflow, *n* (%)	
ATS	30 (52.6%)
ATP	27 (47.4%)

*Abbreviations:* ART, adaptive radiotherapy; ATP, adapt to position; ATS, adapt to shape; GTR, gross total resection; HGG, high‐grade glioma; IDH, isocitrate dehydrogenase; LGG, low‐grade glioma; MGMT, O6‐methylguanine‐DNA methyltransferase; STR, subtotal resection; TERT, telomerase reverse transcriptase.

### 3.1 Pre‐Treatment Tumor Changes

**FIGURE 2:**
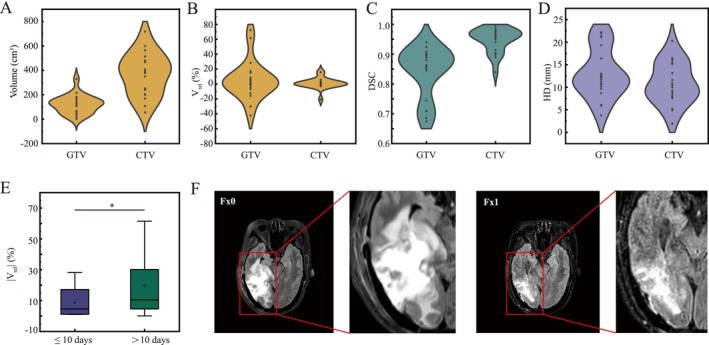
Summary of pre‐treatment changes in gross tumor volume (GTV) and clinical target volume (CTV). (A) Absolute volumes of GTV and CTV at Fx0. (B) Relative volume changes of GTV and CTV at Fx1. (C) Dice similarity coefficient (DSC) of GTV and CTV at Fx1. (D) Hausdorff distance (HD) of GTV and CTV at Fx1. (E) The volume changes in GTV at different intervals from simulation to Fx1. (F) Serial changes in tumor area for a representative patient at Fx0 and Fx1.

Evaluate pre‐treatment tumor changes by comparing the target between Fx1 and Fx0 (Figure 2). The median absolute volume of GTV and CTV for the overall cohort at Fx0 was 129.31 cm^3^ (range: 3.82–329.76 cm^3^) and 384.68 cm^3^ (range: 55.63–717.83 cm^3^) (Figure 2A). The median absolute volume of GTV and CTV at Fx1 was 125.81 cm^3^ (range: 6.58–357.93 cm^3^) and 384.43 cm^3^ (range: 57.45–709.96 cm^3^) (Figure 2A). The results show that the median pre‐treatment V_rel_, DSC, and HD of GTV were 2.13% (range: −42.27%–72.25%), 0.87 (range: 0.67–0.94), and 12.22 mm (range: 3.76–22.24 mm), respectively (Figure 2B–D). Additionally, the median pre‐treatment V_rel_, DSC, and HD of CTV were 0.86% (range: −20.71%–15.93%), 0.96 (range: 0.84–0.99), and 10.49 mm (range: 1.93–20.28 mm), respectively (Figure 2B–D).

No statistically meaningful differences were observed in tumor changes with MGMT methylation status, as well as those with IDH and TERT mutation status. The interval from surgery to Fx0 was not associated with pre‐treatment changes. In contrast, the interval from simulation to Fx1 was associated with pre‐treatment tumor changes. The short‐interval group (≤ 10 days) included 25 patients and the long‐interval group (> 10 days) included 32 patients. As detailed in Figure 2E, relative to Fx0, the changes were statistically different for patients with a longer interval (> 10 days) from simulation to Fx1 (*p* < 0.001).

### 3.2 Tumor Dynamics During Treatment

**FIGURE 3:**
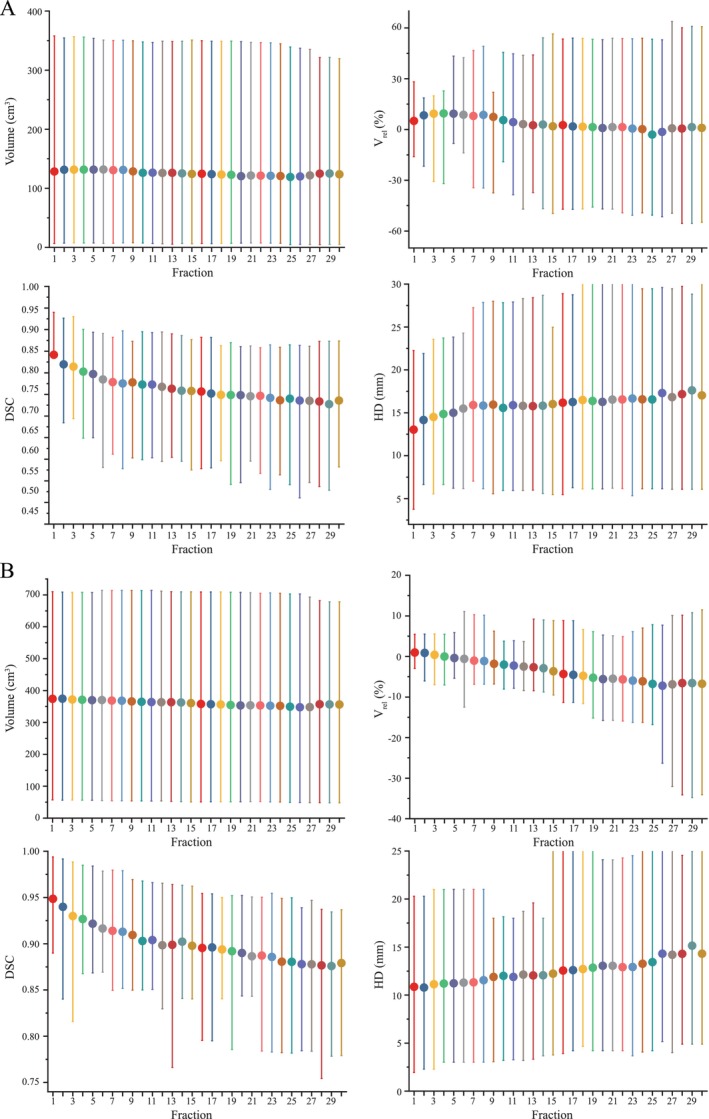
Summary of inter‐fractional changes for gross tumor volume (GTV) and clinical target volume (CTV). (A) boxplot showing the absolute volume, relative volume, Dice similarity coefficient (DSC), and Hausdorff distance (HD) of GTV. (B) boxplot showing the absolute volume, relative volume, Dice similarity coefficient (DSC), and Hausdorff distance (HD) of CTV.

For each fraction, the GTV and CTV on session MRI were evaluated in this study and summarized in Figure 3. The median inter‐fractional V_rel_, DSC, and HD of GTV were 3.28% (range: −76.04%–78.48%), 0.81 (range: 0.36–0.94), and 15.01 mm (range: 3.76–30.28 mm), respectively, when compared with the baseline at Fx0 (Figure 3A). The median inter‐fractional V_rel_, DSC, and HD of CTV were −2.32% (range: −34.78%–25.17%), 0.91 (range: 0.72–0.99), and 11.76 mm (range: 1.93–28.24 mm), respectively (Figure 3B). From the overall results, the volume and DSC decreased and the HD increased during treatment. These results indicate that tumor changes became increasingly apparent during treatment. Notably, alterations in the tumor were observed during the second week of treatment (Fx6–10).

### 3.3 Correlation of Tumor Dynamic and Covariables

**FIGURE 4:**
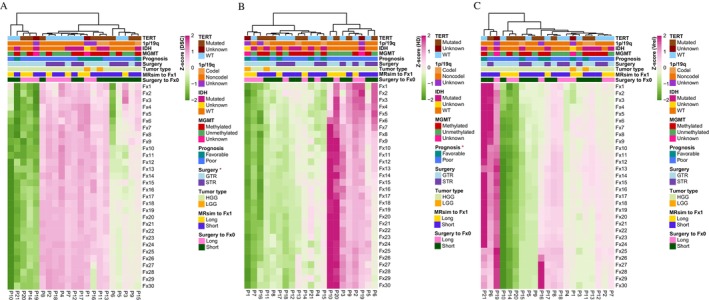
Correlation of tumor dynamic and covariables. (A) Heatmap showing the association of the DSC with covariables. (B) Heatmap showing the association of the HD with covariables. (C) Heatmap showing the association of the V_rel_ with covariables.

In this study, we analyzed the association of the DSC, HD, and V_rel_ with covariables such as extent of surgical resection, MGMT, time from surgery to Fx0, time from simulation to Fx1, IDH, TERT, 1p/19q status, and prognosis. The results were all negative except for extent of surgical resection and prognosis (Figure 4). The current data indicated that clinical factors and molecular characteristics were not significantly associated with tumor dynamics (Figure 4A–C). Notably, given the relatively limited sample size of the present study, cautious interpretation of the study findings is warranted to ensure scientific rigor. Further investigation with a larger cohort is necessary to validate and expand upon these results. Furthermore, a notable association was observed between surgical resection and the DSC, as well as between prognosis and the HD (Figure 4A,B).

### 3.4 Application of Online ART and Dosimetric Advantages

**FIGURE 5:**
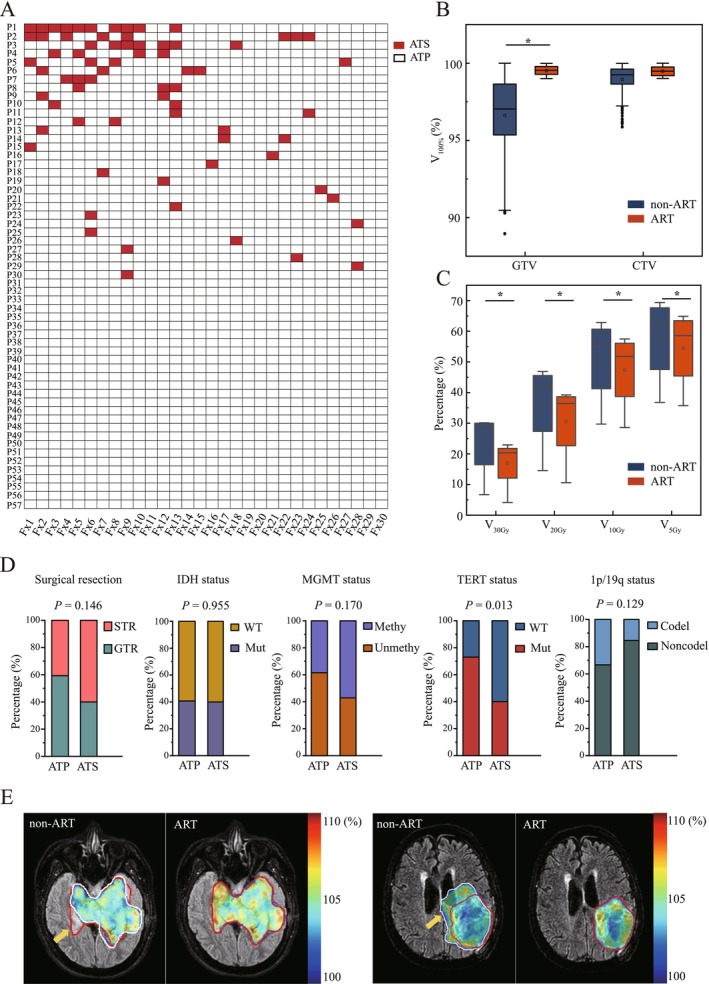
Application of online ART and dosimetric comparison between ART and non‐ART plans. (A) The application of adapt‐to‐shape (ATS) and adapt‐to‐position (ATP) approaches. (B) The dose coverage for GTV and CTV between ART and non‐ART plans. (C) The dose differences for normal brain tissue between ART and non‐ART plans. (D) The association of the two ART approaches with covariables. (E) Illustrative representative changes between the ART and non‐ART plans. The pre‐treatment and daily GTV were contoured by white and red lines, respectively.

Figure 5A presents the implementation details of ATS and ATP. For improved visual clarity of individual patient treatments, the entities on the Y‐axis are ordered according to the descending count of ATS interventions. Among the 57 patients treated with MR‐Linac, MRIgOART using ATS was administered at least once during treatment in 52.6% of patients (30/57). Sixty percent (18/30) of patients underwent their first ATS procedure during the period from Fx1 to Fx10. There were 26.7% of patients (8/30) who received more than three ATS fractions, with 20% of patients (6/30) treated with two ATS fractions and 53.3% of patients (16/30) treated with one ATS fraction. In particular, one patient received ten ATS fractions during treatment. The 47.4% of patients (27/57) underwent all treatment fractions using the ATP workflow. For the 1710 treatment fractions in this study, 71 fractions used ATS workflow. There were 73.2% (52/71) ATS fractions that occurred in Fx1–14, of which at least 3 patients were treated with ATS at each fraction.

Additionally, we further examined the association of the two ART approaches with covariables such as extent of surgical resection, MGMT, IDH, TERT, and 1p/19q status (Figure 5D). All results were negative except for the TERT status. We found that patients with TERT wild‐type had a higher frequency of ATS implementation (*p* = 0.013).

Figure 5B shows the dosimetric differences between ART and non‐ART plans for GTV and CTV. For 71 ATS fractions, we simulated the non‐ART plans generated by recalculating from reference plans. For the criteria with V_100%_ prescription dose, GTV coverage for ART and non‐ART plans was 99.53% ± 0.29% and 96.61% ± 2.67%, and CTV coverage for ART and non‐ART plans was 99.29% ± 0.23% and 98.94% ± 1.05%, respectively. A statistically observed improvement in GTV coverage was observed using online ART plans in ATS fractions (*p* < 0.001). Furthermore, a comparative analysis was conducted to evaluate the irradiation dose administered to normal brain tissue between ART plans and non‐ART plans (Figure 5C). The V_5Gy_, V_10Gy_, V_20Gy_, and V_30Gy_ of normal brain for non‐ART plans were 59.98% ± 14.61%, 51.77% ± 14.75%, 36.46% ± 14.85%, and 23.23% ± 11.17%, respectively. Additionally, the V_5Gy_, V_10Gy_, V_20Gy_, and V_30Gy_ of normal brain for ART plans were 56.82% ± 13.13%, 48.19% ± 13.04%, 30.65% ± 13.48%, and 16.93% ± 8.59%, respectively. In comparison to non‐ART treatment plans, the ART plans delivered a lower irradiation dose to normal brain tissue (*p* < 0.001), as shown in Figure 5C. Representative examples illustrating differences are displayed in Figure 5E. The left case required online ART due to an increase in the tumor volume during treatment. Conversely, the right case used online ART to reduce radiation exposure to normal brain tissue, owing to a reduction in the tumor volume during treatment.

### 3.5 Oncological Outcome and Patterns of Failure

**FIGURE 6:**
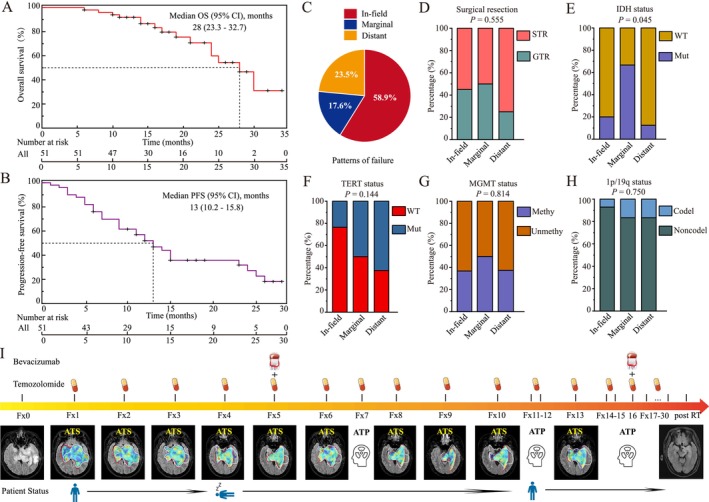
Survival analyses of HGG patients who received MRIgOART. Kaplan–Meier survival curves for overall survival (OS) (A) and progression‐free survival (PFS) (B). (C) The proportion of patterns of failure. (D), (E), (F), (G), (H). The association of the patterns of failure with covariables. (I) The treatment protocol outlines the administration of both adaptive radiotherapy (ART) and concomitant therapy, along with corresponding patient status assessments. The pre‐treatment and daily GTV were contoured by white and red lines, respectively.

At the data cutoff, fifty‐one patients diagnosed with HGG were enrolled in the efficacy analysis. As shown in (Figure 6A,B), after a median follow‐up of 16 months (interquartile range, 12.5–23.5 months), MRI based tumor progression developed in 34 of 51 patients. The median PFS was 13 months (95% CI, 10.2–15.8 months). The 1‐year PFS rate was determined to be 53.1% (95% CI, 38.1%‐66%) in the study cohort. At the time of data cut‐off, 15 of 51 patients (29.4%) had died. The median OS was 28 months (95% CI, 23.3–32.7 months). The 1‐year OS rate was 91.9% (95% CI, 79.8%‐96.9%).

The proportion of patterns of failure is summarized in Figure 6C. Of the thirty‐four documented recurrences, 20 patients (58.9%) had in‐field failure, 6 patients (17.6%) had marginal failure, and 8 patients (23.5%) had distant failure. We further examined the association of the patterns of failure with covariables such as extent of surgical resection, MGMT, IDH, TERT, and 1p/19q status (Figure 6D–H). The results were all negative except for the extent of IDH status. The ratio of wild‐type IDH was higher in both the in‐field and distant failure groups compared to the marginal failure group.

In principle, adaptive cancer therapy represents the most feasible strategy for managing the adaptive dynamics of cancer through the combination of drug therapies and radiotherapy. As shown in Figure 6I, the standard treatment regimen for this patient is maximal safe surgical resection followed by concurrent 30 fractions of radiotherapy and TMZ chemotherapy for six weeks in this study. During the Fx4 treatment, the patient experienced tumor progression or cerebral edema, accompanied by manifestations of disturbed consciousness. Intravenous bevacizumab (500 mg/Q2W) was administered concurrently with ongoing radiotherapy. The patient's condition improved after the Fx10 treatment session, enabling successful completion of radiotherapy. The tumor also shrank significantly by the conclusion of the treatment.

## 4 Discussion

Following surgical resection of brain glioma, radiotherapy serves as the standard therapeutic approach. Compared to the conventional CBCT‐guided radiotherapy, the MRI‐guided radiotherapy can monitor tumor dynamics and correct the anatomical changes by the high soft‐tissue resolution session MRI during treatment. Although MRI‐guided radiotherapy shows potential for treating brain tumors, a recent review indicated that only a limited number of centers utilized MR‐Linac for managing patients with such tumors [15]. This is because MR‐Linac is commonly used for tumors located in the torso [16, 17, 18] that move between or during treatments due to physiological motion. Nevertheless, several studies have indicated that the dynamic changes of brain glioma vary significantly among different patients, and notable morphologic alterations can occur even when the target volume is shrinking [6, 19]. Our study was designed to investigate the variations of brain glioma during treatment and evaluate the potential benefits of MRIgOART in brain glioma. This study first reported the preliminary outcomes of MRIgOART in HGG patients.

Firstly, we evaluated the pre‐treatment tumor changes defined the differences between Fx0 and Fx1. A few studies focused on the pre‐treatment changes in brain glioma. However, this time interval is often influenced by factors such as the patient's condition, treatment procedures, equipment availability, and holidays. Additionally, the tumor may undergo changes during this period. The obvious pre‐treatment changes in GTV were observed (Figure 2F); however, marked interindividual variability was notably evident. No significant associations were detected between the changes and factors such as MGMT status, IDH mutation, TERT mutation, or the interval between surgery and Fx0 in this cohort. As detailed in Figure 2E, patients with an interval longer than 10 days from simulation to the Fx1 exhibited more significant tumor changes (*p* < 0.001). The results indicated that patients with an interval of more than 10 days between simulation and Fx1 may derive greater potential benefits from MRIgOART. The 7% (4/57) patients were treated by ATS in first fraction in this study. For conventional image‐guided radiotherapy (IGRT), the time from simulation to Fx1 should also be within 10 days.

A recent study using standalone MRI to quantify inter‐fraction dynamics based on sequential MRI scans obtained throughout standard 6‐week chemoRT [19]. During chemoRT for glioblastoma, clinically apparent tumor dynamics were observed. Structural alterations are observed in individuals with glioblastoma who undergo daily imaging with 0.35 T MRI‐linac during the chemoRT course [6]. However, a key challenge in these studies is the limited number of imaging time points conducted. This study firstly used daily MRI monitoring to specifically report on tumor dynamics during concurrent chemoRT. Compared to Fx0, the median inter‐fractional V_rel_, DSC and HD of GTV were 3.28% (range: −76.04%–78.48%), 0.81 (range: 0.36–0.94), 15.01 mm (range: 3.76–30.28 mm), respectively (Figure 2B–D). Guided by these GTV dynamic changes, the CTV presented a similar variation trend. Stewart et al. [19] also found that, at some stage during chemoRT, 58% of patients had GTV and CTV migrations over 5 mm, 24% had migrations beyond 10 mm, and 8% had migrations in excess of 15 mm, respectively. In general, the volume of the tumor gradually reduced throughout the treatment period, while alterations in its position and shape grew more prominent as time went on. Additionally, these findings are broadly consistent with those reported by Mehta et al., who noted a general reduction in target volume during radiotherapy and increased edema in 1 of 4 patients within a preliminary cohort treated with the ViewRay system (Oakwood Village, OH) [20]. Additionally, our results showed that the most apparent tumor changes were always observed in the second treatment week (Fx6–10). The results suggest that oncologists should closely monitor tumor changes between Fx6 and Fx10. For conventional IGRT, the MRI may also be received in the second treatment week to decide whether to modify the treatment plan. Furthermore, a notable association was observed between surgical resection and the DSC, as well as between prognosis and the HD (Figure 4). Other clinical factors and molecular characteristics do not differ significantly among tumor dynamics, indicating that tumor behavior varies individually. However, our results were limited by the small dataset. A larger sample size may uncover underlying association between tumor dynamics and clinical as well as molecular characteristics.

Collectively, the tumor dynamic data indicated that personalized tumor monitoring and adaptive treatment plan adjustment hold potential as a research direction worth pursuing. Conventional IGRT for brain glioma radiotherapy relies on isotropic margin expansion to generate the PTV, ensuring the accurate delivery of treatment. The geometric framework is designed to accommodate tumor changes and motion, as well as the margin accounting for potential image‐verification and setup errors. Landmark research focusing on evaluating radiation therapy outcomes, conducted by the European Organization for Research and Treatment of Cancer and the Radiation Therapy Oncology Group, described their treatment volume via a single‐ or two‐phase method specifically, the GTV paired with a 2‐ to 3‐cm expansion to the field edges [1, 21]. In contrast, a more modern approach typically defines the PTV as the CTV with a 3‐ to 5‐mm expansion margin [22, 23]. In addition to covering the at‐risk peritumoral region, such large volumetric expansions are likely responsible for most inter‐fractional variations in tumor dynamics. However, these approaches cannot perfectly ensure target coverage. Meanwhile, they may lead to the irradiation of a considerable volume of healthy brain tissues. When compared to the pre‐treatment MRI scans, Manon et al. [8] noted that 80% of the target volumes outlined on mid‐treatment scans would have resulted in a geographic miss on the subsequent boost plan, assuming no changes in tumor volume. Furthermore, 27% of cases were classified as a complete miss, defined as the portion of the tumor that extended more than 2 cm beyond the original GTV delineation.

Currently, some studies focused on identifying and quantifying these tumor dynamic changes to select appropriate treatment margin [19]. However, the MRIgOART makes it possible to create online ART plans that are optimized according to the anatomical structures and contours shown in daily MR images. This study firstly reports the application of MRIgOART in brain glioma treatment. For non‐ART plans, the prescribed dose coverage of GTV was less than 99%, while failing target coverage constraints. The online ART plans in ATS fractions improved GTV coverage significantly (*p* < 0.001) (Figure 5B). Meanwhile, the ART plan delivered a lower radiation dose to the normal brain tissue (*p* < 0.001), as shown in Figure 5C. Exposure of the brain to radiation is linked to neurotoxic adverse outcomes such as radionecrosis and impairments in cognitive function [24]. It is widely recognized that the volume of irradiated brain tissue is closely associated with the development of these complications [25]. Theoretically, targeting a smaller volume of brain tissue can help alleviate these adverse effects. If the tumor enlarges, MRIgOART technology can correct deformation errors to avoid missing the target. Conversely, if the tumor shrinks, MRIgOART can adjust the treatment plan to reduce the irradiation area and minimize radiation exposure to normal brain tissues (Figure 5E).

In the workflow of Unity MR‐Linac, the ATS can correct changes in tumor and organ positioning, while the ATP can address patient setup errors and modest anatomical variations. Through process optimization, the durations of ATP and ATS treatments have been reduced to 13 and 20 min, respectively. This improvement has been accepted by doctors, physicists, dosimetrists, and patients. Figure 5 illustrated the ATS implementation. We found that the frequencies of ATS were higher in wild‐type than in mutant type of TERT (*p* = 0.013). We also found that the ratio of ATS was higher in STR compared to GTR. These results suggested that patients with the wild‐type of TERT or STR experience more apparent tumor changes during treatment. Consequently, these patients may derive greater benefits from MRIgOART.

Despite advances in surgical techniques, neuro‐imaging, molecular profiling and systemic therapies, the RT component has seen little evolution over the past several decades [26]. Specifically, the RT volume fails to incorporate advancements in image‐guided and radiation delivery technologies. Notably, the delineation of the GTV and CTV frequently relies on postoperative MRI, which only provides a static snapshot of the tumor and its surrounding anatomical structures before the initiation of treatment. For conventional CBCT‐guided radiotherapy, the Glioblastoma Longitudinal Imaging Observational (GLIO) prospective serial imaging study conducted by Ong et al. [27] showed that median PFS and OS were 8.5 months and 20.4 months for 129 GBM patients treated between 2016 to 2021 with 60 Gy/30 fractions with TMZ and standard margins using CBCT‐Linac image‐guidance. Buchroithner [28], Sampson [29], Weller [30], Wen [31] and Wheeler [32] trials reported the efficacy of chemo‐radiotherapy alone for the treatment of patients diagnosed glioblastoma. The median PFS and OS were 6.3 ~ 9 months and 13.7 ~ 20 months, respectively. A comprehensive review of the literature about HGG by Ashby et al. reported that the weighted mean of median OS was 18.2 months (ten trials, *n* = 379, range 12.7 to 21.3 months), and the weighted mean of median PFS was 9.7 months (seven trials, *n* = 287, range 7 to 12.9 months) [33].

The integration of MR‐Linac's high‐resolution imaging capabilities enables MRIgOART to not only correct tumor deformation errors during delivery but also facilitates real‐time assessment of treatment response, allowing for dynamic optimization of concomitant therapy protocols. Instead, we will leverage the specific example in Figure 6I to illustrate how real‐time MRI guidance enables a comprehensive adaptive treatment approach, allowing for dynamic integration and adjustment of all concurrent therapies (including targeted agents), thus showcasing a synergistic potential beyond solely physical dose precision. This therapeutic modality holds promise for potentially enhancing survival outcomes. The study was the first to report the PFS and OS of MRIgOART for patients with HGG (Figure 6A,B). At data cutoff, fifty‐one patients with HGG were included in the efficacy analysis. Our results compare favorably with previous reports, the median PFS was 13 months (95% CI, 10.2–15.8 months). The 1‐year PFS rate was 53.1% (95% CI, 38.1%‐66%). The median OS was 28 months (95% CI, 23.3–32.7 months). The 1‐year OS rate was 91.9% (95% CI, 79.8%‐96.9%).

In the present study, our data showed that 58.9% of tumor recurrences occurred within the irradiated field, 17.6% were identified as marginal recurrences, and the remaining 23.5% were distant recurrences (Figure 6C). Several research investigations have examined individuals who underwent radiation therapy before the advent of TMZ, revealing that the primary mode of treatment failure is mainly confined to the original site. Gebhardt's study demonstrated that 81% of patients experienced in‐field recurrences [34]. Another study of the patterns of failure of GBM after chemoRT was published by Brandes et al. [35]. They reported 72.2% of recurrences in the radiation field. Compared to the previous reports, our study's preliminary results suggest that MRIgOART may potentially reduce the rate of recurrences in the radiation field. We also found the association of the patterns of failure with IDH status (Figure 6E). The ratio of wild‐type IDH was higher in in‐field and distant failures compared to marginal failures.

In this study, MRIgOART presents the tumor dynamics and potential clinical advantages. The limited number of participants and brief follow‐up period could influence the extent to which the findings can be applied to a broader population. Furthermore, the study did not include a control group and had a retrospective design. These findings should be verified by a larger randomized trial of MRIgOART versus conventional IGRT. Currently, this work is actively being conducted at our center. Additional in‐depth investigations remain necessary, including identifying the trigger conditions for online ART, understanding the guidelines for adjusting target volumes, and establishing methods to ensure precise dose accumulation in tumors that are shrinking. The prolonged treatment duration of MRIgOART may add to the challenges in clinical practice, and its practical implementation remains a demanding task in a busy cancer center. The MRIgOART process harbors considerable potential for further optimization across multiple key domains. The process of treatment will be refined through the integration of advanced technologies, such as VMAT [36], AI‐based treatment planning [37] and contouring [38], which will contribute to the benefit of MRIgOART. Furthermore, the functional imaging strategies, including dynamic contrast‐enhanced (DCE) perfusion, diffusion‐weighted imaging (DWI), and chemical exchange saturation transfer (CEST), have been rigorously validated to be feasible and applicable on the MR‐Linac [39, 40]. These advanced functional MRI techniques are capable of characterizing features closely associated with tumor radiosensitivity and radioresistance, including the biophysical, microstructural, cellular, and metabolic properties of tumors. A core question centers on how to integrate state‐of‐the‐art imaging technologies into target delineation and incorporate them into individualized adaptive treatment regimens. The adaptive radiotherapy based on physiologic changes during RT is a subject of further study [26].

## 5 Conclusions

Apparent tumor dynamics were observed during chemoRT in patients with brain glioma. Our study suggests that MRIgOART offers a promising advancement in the management of brain glioma. MRIgOART not only corrects daily anatomical variations but also enables real‐time assessment of treatment response, thereby facilitating dynamic optimization of concomitant therapy protocols. Preliminary findings in patients with HGG suggest a lower incidence of in‐field recurrences and indicate promising outcomes associated with MRIgOART. However, larger randomized trials are required to validate these findings and further refine the corresponding treatment strategies.

## Author Contributions

S.D. contributed to conceptual design, data analysis and writing the manuscript; X.Y. and M.S. contributed to data analysis; Y.C. contributed to data analysis, conceptual design, and writing the manuscript; H.L., M.Q. and X.H. contributed to data analysis and provided important intellectual input; Y.W., B.L., Y.Z., X.D., M.D., S.D., and Y.M. collected data and provided patient samples; Y.M., W.H. and Z.L. contributed to conceptual design and project integrity. All authors read and approved the final manuscript.

## Funding

This work was supported by National Natural Science Foundation of China, 12405409, 62271475, 82102877. Fundamental Research Funds for the Central Universities, Clinical Research 5010 Program, Sun Yat‐sen University, 2022008.

## Ethics Statement

The research was approved by the Sun Yat‐sen University Cancer Center Ethics Review Board (B2024‐857‐01).

## Conflicts of Interest

The authors declare no conflicts of interest.

## Data Availability

The data that support the findings of this study are available from the corresponding author upon reasonable request.
